# Effects of 3-Month Astaxanthin Supplementation on Cardiac Function in Heart Failure Patients with Left Ventricular Systolic Dysfunction-A Pilot Study

**DOI:** 10.3390/nu12061896

**Published:** 2020-06-26

**Authors:** Takao Kato, Takatoshi Kasai, Akihiro Sato, Sayaki Ishiwata, Shoichiro Yatsu, Hiroki Matsumoto, Jun Shitara, Azusa Murata, Megumi Shimizu, Shoko Suda, Masaru Hiki, Ryo Naito, Hiroyuki Daida

**Affiliations:** 1 Department of Cardiovascular Medicine, Juntendo University Graduate School of Medicine, Tokyo 113-8421, Japan; tkatou@juntendo.ac.jp (T.K.); ak-sato@juntendo.ac.jp (A.S.); s-ishiwata@juntendo.ac.jp (S.I.); syatsu@juntendo.ac.jp (S.Y.); hmatsumo@juntendo.ac.jp (H.M.); jshitara@juntendo.ac.jp (J.S.); azmurata@juntendo.ac.jp (A.M.); megumi-s@juntendo.ac.jp (M.S.); ssuda@juntendo.ac.jp (S.S.); ma-hiki@juntendo.ac.jp (M.H.); rnaitou@juntendo.ac.jp (R.N.); daida@juntendo.ac.jp (H.D.); 2 Cardiovascular Respiratory Sleep Medicine, Juntendo University Graduate School of Medicine, Tokyo 113-8421, Japan; 3 Sleep and Sleep-Disordered Breathing Center, Juntendo University Hospital, Tokyo 113-8421, Japan

**Keywords:** antioxidant, left ventricular ejection fraction, oxidative stress, 6-min walk test

## Abstract

Astaxanthin has strong antioxidant properties. We conducted a prospective pilot study on heart failure (HF) patients with left ventricular (LV) systolic dysfunction to investigate improvements in cardiac function and exercise tolerance in relation to suppression of oxidative stress by 3-month astaxanthin supplementation. Oxidative stress markers—serum Diacron reactive oxygen metabolite (dROM), biological antioxidant potential (BAP), and urinary 8-hydroxy-2′-deoxyguanosine (8-OHdG) concentrations, LV ejection fraction (LVEF), and 6-min walk distance (6MWD) were assessed before and after 3-month astaxanthin supplementation. Finally, the data of 16 HF patients were analyzed. Following 3-month astaxanthin supplementation, dROM level decreased from 385.6 ± 82.6 U.CARR to 346.5 ± 56.9 U.CARR (*p* = 0.041) despite no changes in BAP and urinary 8-OHdG levels. LVEF increased from 34.1 ± 8.6% to 38.0 ± 10.0% (*p* = 0.031) and 6MWD increased from 393.4 ± 95.9 m to 432.8 ± 93.3 m (*p* = 0.023). Significant relationships were observed between percent changes in dROM level and those in LVEF. In this study, following 3-month astaxanthin supplementation, suppressed oxidative stress and improved cardiac contractility and exercise tolerance were observed in HF patients with LV systolic dysfunction. Correlation between suppression of oxidative stress and improvement of cardiac contractility suggests that suppression of oxidative stress by astaxanthin supplementation had therapeutic potential to improve cardiac functioning.

## 1. Introduction

Astaxanthin, a carotenoid like β-carotene and lycopene, is widely distributed in nature, especially in deep-red-colored natural sea products such as salmon, salmon roe, shrimp, and crab [1]. As humans cannot synthesize carotenoids, astaxanthin is acquired and accumulated through the diet. After ingestion, astaxanthin mixes with bile acid to form a micelle, is then passively absorbed in the intestine, and enters into systemic circulation via the lymphatic system [2]. Reportedly, astaxanthin has a strong antioxidant effect with potency over 100-fold higher than that of α-tocopherol [3]. Unlike other carotenoids that generally have simultaneous antioxidative and pro-oxidative properties under certain conditions [4], astaxanthin shows solely antioxidative effects [4]. Indeed, several studies of relatively healthy subjects reported that astaxanthin inhibited oxidative stress and systemic inflammatory responses. For instance, astaxanthin supplementation reduced both plasma levels of oxidative stress markers and serum levels of pro-inflammatory cytokines in healthy women [5]. Another study reported reduced oxidative stress markers in overweight and obese subjects [6]. Because enhanced oxidative stress and systemic inflammation are associated with cardiovascular impairments and can result in cardiovascular disease [7,8,9], astaxanthin may have protective effects on the cardiovascular system [7]. However, in a recent clinical trial involving renal transplant recipients with enhanced oxidative stress and inflammation, astaxanthin failed to produce any improvement of those vascular parameters [10]. Other data have suggested that astaxanthin—due to its antioxidant properties—has beneficial effects on cardiac function and tolerance of strenuous exercise [11,12]. In addition, one human study showed that astaxanthin supplementation improved exercise performance in competitive athletes [13]. These findings suggest that astaxanthin supplementation is beneficial for cardiac function and/or exercise tolerance, particularly in subjects with enhanced oxidative stress and inflammation such as those with heart failure (HF).

In patients with HF, oxidative stress worsens the progression of HF [9]. Therefore, enhanced oxidative stress can be considered a potential therapeutic target for HF patients [7,8,9]. However, previous clinical trials have not reported positive clinical outcomes after anti-oxidative stress therapy for HF [9]. Because there are no human data regarding the effects of astaxanthin supplementation on oxidative stress, systemic inflammation, cardiac function, and exercise tolerance in HF patients, we conducted a prospective small uncontrolled pilot study to investigate the effects of a 3-month astaxanthin supplementation in HF patients with left ventricular (LV) systolic dysfunction. We hypothesized that 3 months of astaxanthin supplementation would improve LV function and exercise tolerance due to the suppression of oxidative stress.

## 2. Materials and Methods

### 2.1. Subjects

We enrolled patients with systolic HF at Juntendo University Hospital (Tokyo, Japan) if they met the following criteria: men and women aged ≥20 years, patients with HF due to ischemic or non-ischemic cardiomyopathy, patients with an LV ejection fraction (LVEF) <50% on echocardiography, patients labeled as New York Heart Association (NYHA) functional class ≥II, and patients with a stable clinical status evidenced by the absence of symptoms related to the acute exacerbation of HF. Exclusion criteria were as follows: patients who cannot perform a 6-min walk test; those who cannot answer the questionnaire without assistance; current smokers; patients with acute coronary syndrome or that have had cardiac surgery during the previous 4 weeks; patients with organic valvular heart diseases, chronic inflammatory diseases, chronic lung diseases, or malignancies; and patients under dialysis. This prospective observational pilot study (UMIN-ID: 000014088) was approved by the Juntendo University Hospital Institutional Review Board (#14-019), and complied with the ethical principles of the Declaration of Helsinki. Written informed consent was obtained from all participants.

### 2.2. Data Collection

On the morning of the day before starting astaxanthin supplementation, height and weight were measured, body mass index (BMI) was calculated, blood pressure and heart rate were measured, and venous blood samples were obtained after an overnight fast. The estimated glomerular filtration rate (eGFR) based on serum creatinine level was assessed [14]. Plasma concentration of astaxanthin was measured via high-performance liquid chromatography whose details were previously described [15]. In addition to serum C-reactive protein (CRP) levels, tumor necrosis factor-α (TNF-α) levels—an inflammatory marker—were measured using a commercially available human Quantikine HS ELISA kit (R&D systems, Minneapolis, MN, USA). Urinary 8-hydroxy-2′-deoxyguanosine (8-OHdG) concentrations were measured using the urinary oxidative stress marker measurement system, ICR-001 (Immunochromatography method, Techno Medica Co.). Urinary creatinine was also measured, and data were reported as the urinary 8-OHdG (ng/mL)/creatinine (mg/mL) ratio. Assays of oxidative stress in serum were performed with the Diacron reactive oxygen metabolites (dROM) test, which measures the total oxidant capacity of samples relative to the chromogenic substrate N, N-diethylparaphenylendiamin. Total antioxidant capacity was assayed using the biological antioxidant potential (BAP) test, which measures the capacity of a sample to reduce ferric ions to ferrous ions with a thiocyanate derivative as a chromogen. All analyses were performed with a free radical analyzer system (Free Carrio Duo, Wismerll Company Ltd., Tokyo, Japan).

To assess cardiac function, cardiac chamber quantification using 2-dimensional echocardiography was performed according to American Society of Echocardiography guidelines [16] on the day before starting astaxanthin supplementation. The LVEF was calculated according to the modified Simpson method. Images were stored in at least 3 cardiac cycles, and the final values represented the average of at least 3 measurements. All echocardiographic studies were performed and interpreted by experienced cardiologists who were blinded to the details of the present study and other clinical data of participants. Sonographers were blinded to study enrollment and were not involved in the present study. Lastly, the plasma level of B-type natriuretic peptide (BNP) was measured, and, to quantify exercise tolerance, the 6-min walk test was performed according to established methods; the total distance walked in 6 min (6MWD) was recorded [17].

### 2.3. Astaxanthin Supplement and Study Protocol

In this prospective small uncontrolled pilot study, once daily for 3 months, patients orally ingested a commercially available astaxanthin supplement containing 12 mg of astaxanthin, 40 mg of tocotorienol, and 30 mg of L-ascorbic acid 2-glucoside (AstaReal ACT, AstaReal Co., Ltd., Tokyo, Japan). The patients visited our HF clinic every month, and no changes in the other medications were allowed during the study period. Measurements of BMI, blood pressure, and heart rate were performed at every clinic visit. Blood and urine sampling, echocardiography, and 6-min walk test were repeated at a follow-up visit 3 months after starting astaxanthin supplementation.

### 2.4. Statistical Analyses

Because there were no data regarding the effects of astaxanthin supplement on patients with HF, we did not calculate a specific sample size and instead conducted the present study as a pilot study. Nevertheless, we planned to perform analyses once ≥15 patients had completed their 3-month follow-up period. Values are expressed as mean ± SD for normally distributed data or median [interquartile range] for non-normally distributed data. Correlations between the baseline HF-related parameters (i.e., LVEF, plasma BNP levels, and 6MWD) and oxidative stress/inflammatory markers (i.e., dROM, BAP, 8-OHdG, CRP, and TNF-α concentrations) were assessed using the Pearson and Spearman correlation coefficients for normally and non-normally distributed data, respectively. Differences between baseline and follow-up measurements were compared using paired t-test for normally distributed data or Wilcoxon signed-rank test for non-normally distributed data. Changes in parameters from baseline to 3 months (i.e., Δ) were calculated as Δ = [(values at 3 months) − (values at baseline)] and expressed as %Δ [Δ/(values at baseline) × 100] except for plasma astaxanthin concentration because astaxanthin was not detected at baseline in most participants. Relationships across changes or percent changes in parameters were assessed using Pearson correlation coefficient for normally distributed data and using Spearman correlation coefficient for non-normally distributed data. A *p* value < 0.05 indicated statistical significance. Analyses were performed by SPSS 23.0 (IBM Corp., Armonk, NY, USA).

## 3. Results

### 3.1. Baseline Patient Characteristics

**Figure 1:**
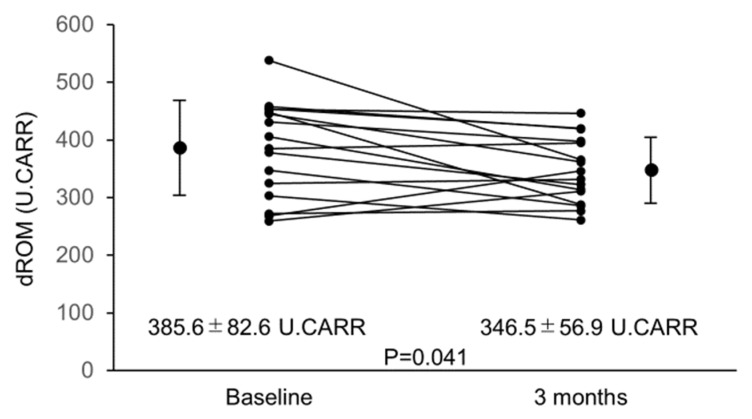
Changes in dROM from baseline to 3 months. dROM decreased significantly from baseline to 3 months after starting astaxanthin supplementation. Abbreviations: dROM, diacron reactive oxygen metabolites.

**Figure 2:**
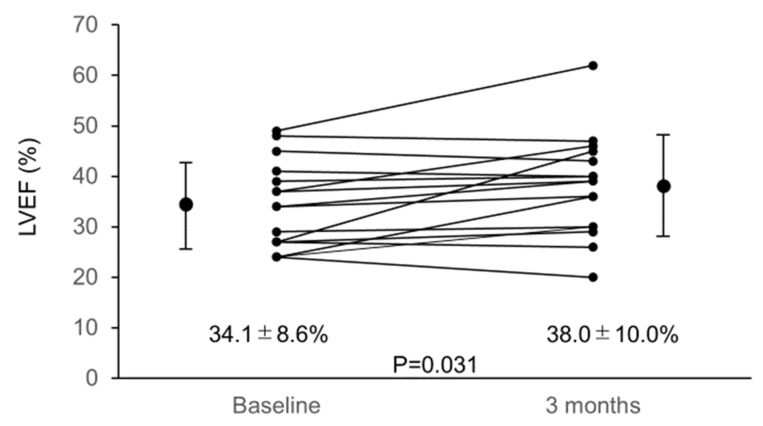
Changes in LVEF from baseline to 3 months. LVEF increased significantly from baseline to 3 months after starting astaxanthin supplementation. Abbreviations: LVEF, left ventricular ejection fraction.

**Figure 3:**
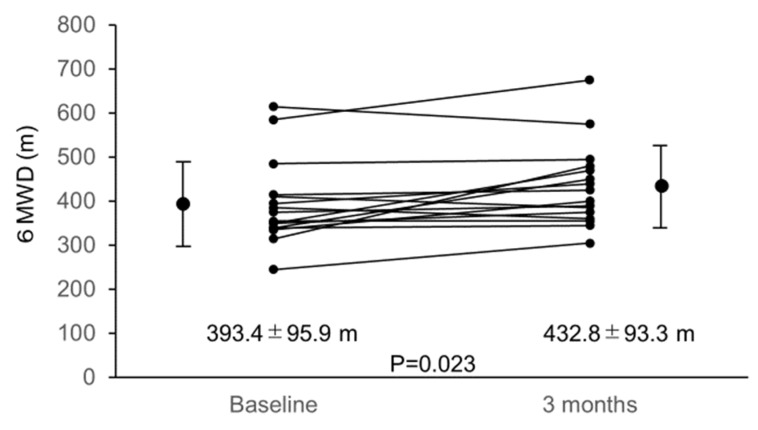
Changes in 6MWD from baseline to 3 months. 6MWD increased significantly from baseline to 3 months after starting astaxanthin supplementation. Abbreviations: 6MWD, 6-min walk distance.

Overall, 19 eligible patients were enrolled, all of whom had been diagnosed as having HF and treated for at least 6 months at enrollment. All the other medications remained unchanged during the study period. Three patients were excluded, two because of lost follow-up records and one because of worsening spinal stenosis at follow-up that caused difficulty during the 6MWD test. Thus, data of 16 patients were analyzed. Characteristics of these patients are summarized in Table 1. Baseline data regarding echocardiography, urinary and blood samples are shown in Table 2 and Figure 1, Figure 2 and Figure 3. A significant correlation was found between the baseline dROM level and LVEF (*r* = −0.516; *p* = 0.041), whereas no such correlations were observed between the baseline dROM and BNP levels or 6MWD (*r* = 0.279, *p* = 0.253 and r = −0.345, *p* = 0.053, respectively). In addition, no significant correlations were found between the other oxidative stress/inflammatory markers (i.e., BAP, 8-OHdG, CRP, and TNF-α concentrations) and the baseline HF-related parameters (i.e., LVEF, plasma BNP level, or 6MWD).

From baseline to after 3 months of astaxanthin supplementation, blood levels of astaxanthin increased significantly from 0 [0] ng/mL to 133.7 [122.1] ng/mL (*p* < 0.001). No complaints or adverse events related to astaxanthin administration were observed. From baseline to 3 months, dROM decreased significantly (Figure 1).

LVEF and 6MWD significantly increased following 3-month astaxanthin supplementation (Figure 2 and Figure 3).

However, the values of the other parameters did not change after the 3-month supplementation period (Table 2).

### 3.2. Relationships between the Baseline dROM Level and the %Δ in Inflammatory and Oxidative Stress Markers, and between the %Δ in LVEF and 6MWD from Baseline to 3 Months

**Figure 4:**
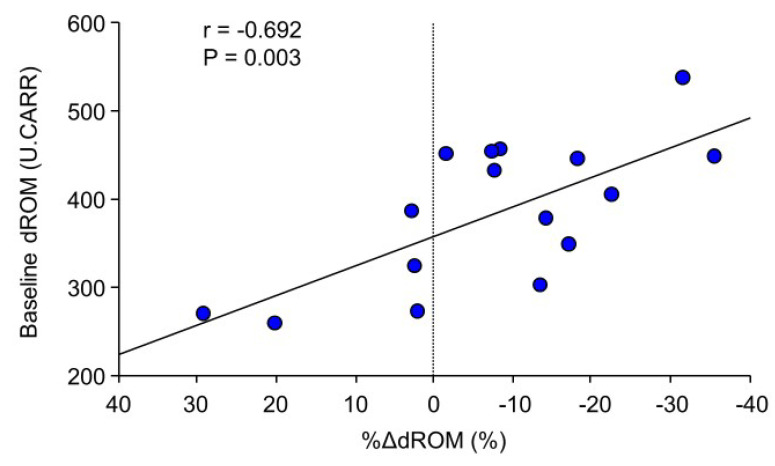
Correlation between the baseline dROM level and the %Δ in dROM level from baseline to 3 months. The greater the baseline dROM level, the greater the reduction in dROM level.Abbreviations: dROM, Diacron reactive oxygen metabolites.

A significant correlation was found between the baseline dROM level and the %Δ in dROM level (Figure 4), whereas no correlations were observed between the baseline dROM level and the %Δ in LVEF and 6MWD (*r* = 0.319, *p* = 0.228 and *r* = −0.264, *p* = 0.323, respectively).

### 3.3. Relationships between Δ in Astaxanthin Levels, %Δ in Inflammatory and Oxidative Stress Markers, and %Δ in Cardiac Functions from Baseline to 3 Months

Correlation coefficients and *p*-values regarding relationships between Δ in astaxanthin levels, %Δ in inflammatory and oxidative stress markers, and %Δ in cardiac functions from baseline to 3 months are shown in Table 3. Significant correlations were observed between %Δ in dROM level and %Δ in LVEF, LV end-diastolic volume index, and LV end-systolic volume index.

## 4. Discussion

Findings of the present prospective small uncontrolled study provide several novel insights into the effects of astaxanthin supplementation on patients with HF. First, augmented baseline oxidative stress level as expressed by increased dROM level was associated with impaired LV systolic function at baseline. Second, 3-month daily oral administration of a commercially available astaxanthin supplement significantly increased the blood concentration of astaxanthin in the HF patients. Third, the dROM results showed a decrease in blood oxidative stress after the 3-month astaxanthin supplementation, whereas the 8-OHdG results showed no decrease in urinary oxidative stress. Fourth, the higher the baseline oxidative stress level, the greater the reduction of blood oxidative stress. Fifth, the serum inflammatory markers did not change after the supplementation. Sixth, improved LV systolic function was observed after the 3-month supplementation. Seventh, exercise tolerance improved after the 3-month supplementation. Finally, significant correlations were observed between the reduction in oxidative stress and the improvement in both the LV systolic function and degree of LV remodeling. These findings suggest that 3-month astaxanthin supplementation improves LV systolic function and exercise tolerance possibly due to the reduction in oxidative stress.

Following 3-month oral administration of a commercially available astaxanthin supplement containing 12 mg of astaxanthin, plasma concentration of astaxanthin was 133.7 [122.1] ng/mL. This value is comparable to the plasma astaxanthin concentration observed in a previous study in which plasma concentration of astaxanthin was measured following the 3-week oral administration of 5 mg and 20 mg of astaxanthin in otherwise healthy overweight and obese subjects [6]. In that study, blood levels of oxidative stress markers decreased significantly with both 5 mg and 20 mg doses of astaxanthin. Thus, administration of 12 mg of astaxanthin with observed plasma concentration in the present study was predicted to potentially decrease blood oxidative stress markers. On the other hand, in the previous study, because different oxidative stress markers were assessed in a different population with different baseline oxidative stress levels, the effects of 12 mg of astaxanthin on the dROM level in our patients with HF may not be applicable in other populations and should be interpreted with caution.

Park et al. reported that astaxanthin supplementation for 8 weeks reduced plasma levels of oxidative stress markers such as 8-OHdG and 8-isoprostane in healthy young women [5]. In addition, Choi et al. reported that astaxanthin supplementation for 3 weeks suppressed lipid peroxidation—represented by malondialdehyde and 8-isoprostane levels—and enhanced antioxidative activity—represented by superoxide dismutase level and total antioxidant capacity [6]. In the present study, markers of oxidative stress (i.e., blood dROM level and urinary 8-OHdG level) and antioxidative stress (i.e., blood BAP level) were measured because a previous report has shown the possible effects of astaxanthin supplementation on dROM and BAP in mice [18]. Although urinary 8-OHdG levels and BAP levels did not change following 3-month supplementation of astaxanthin for reasons that remain unclear, we found that oxidative stress—as expressed by dROM level—could be suppressed by 3-month supplementation of astaxanthin even in patients with HF. In terms of systemic inflammation, a previous report showed that despite no significant change in serum TNF-α levels, 8 weeks of astaxanthin supplementation reduced serum CRP levels in young healthy women [5]. In the present study, however, neither CRP level nor TNF-α level changed following 3-month astaxanthin supplementation; a possible result of the difference in participant health between the studies (i.e., HF patients versus young healthy women).

In one randomized, placebo-controlled, double-blind clinical trial, effects of astaxanthin on surrogate markers of atherosclerosis and arterial stiffness—including aortic pulse wave velocity, augmentation index, brachial forearm reactivity, and carotid artery intima-media thickness—were assessed in kidney transplant recipients [10]. Such patients have an enhanced state of oxidative stress and inflammation, and are at high risk of atherosclerotic disease. Astaxanthin failed to show any beneficial effects on those vascular parameters [10]. On the other hand, two mouse studies showed that astaxanthin administration attenuated exercise-induced oxidative stress markers in heart and gastrocnemius muscles [11] and resulted in greater cardiac mitochondrial membrane potential and contractility index, when compared to control mice [12]. Such results suggest that the antioxidant properties of astaxanthin may have a beneficial effect on myocardium and skeletal muscles. The results of these experimental animal studies support our findings from human HF patients that astaxanthin may reduce oxidative stress and improve LV systolic function. Furthermore, considering the significant correlations we observed between the baseline LVEF and dROM levels, between the baseline dROM levels and the reduction in dROM levels, and between the reduction in dROM levels and the improvements in LVEF, LVEDVI, and LVESVI, the suppression of oxidative stress by astaxanthin administration may play a key role in improving LV remodeling and dysfunction particularly in patients with reduced LVEF and/or augmented oxidative stress. Astaxanthin could increase mitochondrial respiration, adenosine triphosphate production, and consequently, LV systolic function [19,20,21]. Thus, such direct effect of astaxanthin on mitochondria can improve cardiac energetics and contribute to the increases in LVEF and 6MWD. Recently, two additional mouse studies showed that astaxanthin administration reduced myocardial fibrosis following myocardial infarction [22] and mitigated pressure-load induced cardiac dysfunction and myocardial fibrosis [23]; results possibly involve several pathways not limited to only an oxidative stress suppression pathway.

In terms of exercise tolerance, astaxanthin supplementation may improve 6MWD possibly due to improvement of cardiac function. However, as mentioned earlier, astaxanthin may have a direct effect on skeletal muscles, as evidenced in mice [11]. In a randomized controlled study among competitive cyclists who likely did not have any cardiac dysfunction, astaxanthin supplementation improved their exercise performance [13]. In the present study, no significant correlations were observed between either Δastaxanthin and 6MWD or %ΔdROM level and 6MWD. Despite those results, it should be noted that astaxanthin supplementation may have the potential to improve exercise tolerance even in HF patients because poor exercise tolerance is one of the key features of patients with HF.

Our study is subject to some limitations. First, because this was an observational study without control groups, no causal relationship between astaxanthin supplementation and improvements in cardiac function and exercise tolerance was proven. Second, although we found a significant correlation between the reduction in oxidative stress markers and the improvements in cardiac function, the variability of the oxidative stress measurements over time may play some roles. In addition, the improvement in cardiac function may have initially occurred through a mechanism independent of the suppression of oxidative stress. Further, the reduction in dROM level may have occurred as a result of improved cardiac function. Third, the astaxanthin supplement used in the present study containing 40 mg of tocotorienol and 30 mg of L-ascorbic acid 2-glucoside in addition to 12 mg of the astaxanthin. Because both tocotorienol and L-ascorbic acid 2-glucoside also have antioxidant properties, they may too have the potential to reduce dROM and subsequently improve cardiac function. However, because astaxanthin has a stronger antioxidant effect [3,24], we believe that astaxanthin played the major role in the suppression of oxidative stress and improvement of cardiac functions. Ultimately, these observed effects could be recognized as the result of this particular astaxanthin supplement. Fourth, only 16 patients with both ischemic and non-ischemic cardiomyopathies were enrolled. Thus, the number of participants was small even though the present study was only a pilot study. Because no data regarding the effects of astaxanthin supplementation on oxidative stress status, cardiac function, and exercise tolerance in an actual patient population are available, we need a proof-of-concept study like this. A future randomized, controlled, and preferably double-blind trial with an adequately powered sample size is required to draw a solid conclusion.

## 5. Conclusions

In conclusion, this pilot study found that after 3 months of astaxanthin supplementation, the levels of the oxidative stress markers decreased and improvements were observed in both cardiac contractility and exercise tolerance in the HF patients with LV systolic dysfunction. In addition, improvements in cardiac function correlated significantly with the suppression of oxidative stress, suggesting that suppression of HF-associated oxidative stress by astaxanthin supplementation may have therapeutic potential for improving underlying cardiac dysfunction and impaired exercise capacity. 

## Figures and Tables

**Table 1:** Baseline characteristics.

*N* = 16	
**Age, y.o.**	67.4 ± 14.9
BMI, kg/m^2^	23.3 ± 3.8
Women, *n* (%)	3 (19)
NYHA class II, *n* (%)	15 (94)
III, *n* (%)	1 (6)
LVEF, %	34.1 ± 8.6
Ischemic etiology, *n* (%)	7 (44)
Diabetes mellitus, *n* (%)	4 (25)
eGFR ≤60 mL/min/1.73 m^2^, *n* (%)	10 (63)
Medications	
ACE-Is/ARBs, *n* (%)	15 (94)
Beta blockers, *n* (%)	16 (100)
MR antagonists, *n* (%)	12 (75)
Diuretics, *n* (%)	14 (87)

ACE-I, angiotensin converting enzyme inhibitors; ARB, angiotensin II receptor blockers; BMI, body mass index; eGFR, estimated glomerular filtration rate; LVEF, left ventricular ejection fraction; MR, mineral corticoid receptor; NYHA, New York Heart Association.3.2. Changes in measurements from baseline to 3 months.

**Table 2:** Changes in parameters from baseline to 3 months other than dROM, LVEF, and 6MWD.

*n* = 16	Baseline	3 Months	*p*
Body weight, kg	60.4 ± 9.8	61.3 ± 10.5	0.131
Systolic blood pressure, mmHg	112.5 ± 18.2	109.8 ± 17.4	0.472
Diastolic blood pressure, mmHg	60.3 ± 9.8	62.1 ± 10.5	0.485
Heart rate, /min	70.9 ± 9.5	68.3 ± 11.7	0.286
Serum TNF-α, pg/mL	1.61 ± 0.54	1.65 ± 0.65	0.864
Serum CRP, mg/dL	0.15 [0.20]	0.10 [0.05]	0.437
Plasma BAP, μmol/L	2012.0 [203.5]	2003.5 [240.5]	0.756
Urine ratio of 8-OHdG/Cr, ng/mgCre	27.2 ± 13.8	29.4 ± 9.3	0.179
Plasma BNP, pg/mL	196.9 [255.7]	152.7 [201.0]	0.301
LVEDVI, mL/m^2^	105.7 ± 28.4	101.2 ± 27.5	0.326
LVESVI, mL/m^2^	71.3 ± 26.8	65.1 ± 25.5	0.098
E/e’	18.2 ± 9.4	13.9 ± 7.6	0.134
RVSP, mmHg	34.9 ± 15.4	29.6 ± 9.6	0.133
Inferior vena cava, mm	13.0 ± 4.2	13.1 ± 3.3	0.943

BAP, biological antioxidant potential; BNP, B-type natriuretic peptide; CRP, C-reactive protein; dROM, diacron reactive oxygen metabolites; 8-OHdG, 8-hydroxy-2′-deoxyguanosine; LVEDVI, left ventricular end-diastolic volume index; LVEF, left ventricular ejection fraction; LVESVI, left ventricular end-systolic volume index; RVSP, right ventricular systolic pressure; 6MWD, six-minute walk distance; TNF, tumor necrotic factor.

**Table 3:** Correlation coefficients and *p*-values for relationships between changes in astaxanthin level, inflammatory and oxidative stress markers, and cardiac functions from baseline to 3 months.

	%ΔLVEF	%ΔLVEDVI	%ΔLVESVI	%ΔE/e’	%ΔRVSP	%ΔBNP	%Δ6MWD
Δastaxanthin	0.138 *p* = 0.592	−0.044 *p* = 0.864	−0.053 *p* = 0.838	0.324 *p* = 0.210	0.454 *p* = 0.079	0.097 *p* = 0.707	−0.126 *p* = 0.624
%ΔTNF-α	−0.134 *p* = 0.620	−0.216 *p* = 0.421	−0.119 *p* = 0.662	0.042 *p* = 0.878	−0.395 *p* = 0.130	−0.268 *p* = 0.316	0.441 *p* = 0.088
%ΔCRP	−0.397 *p* = 0.152	0.090 *p* = 0.745	0.237 *p* = 0.393	−0.307 *p* = 0.269	0.062 *p* = 0.823	0.237 *p* = 0.393	−0.230 *p* = 0.407
%ΔdROM	−0.509 *p* = 0.049	0.706 *p* = 0.006	0.774 *p* = 0.003	0.309 *p* = 0.232	0.354 *p* = 0.170	0.276 *p* = 0.284	−0.015 *p* = 0.955
%ΔBAP	−0.021 *p* = 0.936	−0.071 *p* = 0.785	−0.015 *p* = 0.955	−0.362 *p* = 0.161	−0.454 *p* = 0.079	−0.238 *p* = 0.356	0.306 *p* = 0.236
%Δ8-OHdG	0.006 *p* = 0.983	−0.033 *p* = 0.904	−0.004 *p* = 0.989	−0.213 *p* = 0.428	0.102 *p* = 0.706	0.266 *p* = 0.320	0.148 *p* = 0.585

BAP, biological antioxidant potential; BNP, B-type natriuretic peptide; CRP, C-reactive protein; dROM, diacron reactive oxygen metabolites; 8-OHdG, 8-hydroxy-2′-deoxyguanosine; LVEDVI, left ventricular end-diastolic volume index; LVEF, left ventricular ejection fraction; LVESVI, left ventricular end-systolic volume index; RVSP, right ventricular systolic pressure; 6MWD, six minute walk distance; TNF, tumor necrotic factor.
